# Effects of Thickening Agents on the Mucociliary Transport Function: Comparison by the Type of Thickening Agents and the Viscosity of Thickened Water

**DOI:** 10.1007/s00455-024-10704-3

**Published:** 2024-05-22

**Authors:** Erika Matsumura, Kanji Nohara, Hikari Fukatsu, Nobukazu Tanaka, Nami Fujii, Takayoshi Sakai

**Affiliations:** 1 Division for Oral-Facial Disorders, Osaka University Dental Hospital, Osaka, Japan; 2 Department of Oral-Facial Disorders, Osaka University Graduate School of Dentistry, 1-8 Yamadaoka, Suita, Osaka, 565-0871 Japan; 3 Fukatsu Dental Clinic, Mie, Japan

**Keywords:** Thickening agent, Mucociliary transport function, Dysphagia, Aspiration pneumonia, Saccharine dye test, Viscosity

## Abstract

Thickening agents effectively prevent liquid aspiration, but their impact on the ease of discharging aspirated liquids from the trachea remains unclear due to alterations in the physical properties of liquids. This study clarifies the effects of thickening agents, comprising various raw materials, on mucociliary transport function, focusing on the viscosities of thickened waters. The subjects were 23 healthy adults. Five types of saccharin solution were prepared: a solution without a thickening agent, a starch-based nectar-like solution, a starch-based honey-like solution, a xanthan-gum-based nectar-like solution, and a xanthan-gum-based honey-like solution. Using these five types of saccharin solutions randomly, each subject underwent five trials of the saccharine dye test to evaluate the mucociliary transport function of the respiratory tract. The saccharin time was defined as the time from the placement of the saccharin solution on the nasal vestibule of the subject to when the subject reported that they became aware of the sweetness. The saccharin transit times for all samples of thickened water were longer compared to those of water without a thickening agent (*p* < 0.01). A comparison between thickened water samples with different viscosities showed that the saccharin transit time was longer when thickened water samples with high viscosity were prepared using the same thickening agent (*p* < 0.01). This suggests that while thickening reduces aspiration, the use of thickening agents may increase the difficulty in discharging aspirated fluids from the trachea.

## Introduction

Elderly individuals often experience pneumonia, a condition that can lead to death in numerous cases [1, 2]. Aspiration, a major factor in pneumonia development [3], poses a greater risk for patients with high aspiration volumes or frequent episodes. Among the types of aspiration, liquid aspiration was reported to be the most common in the elderly [1], which is known to be effectively prevented by the use of thickening agents [4–7].

However, aspiration pneumonia repeatedly occurs in some patients who are prescribed to take liquids with a thickening agent. In addition, there are patients with dysphagia who do not develop aspiration pneumonia even if they are taking liquids that do not thicken [8, 9]. The presence or absence of thickening agents and the level of viscosity are not necessarily related to the onset of aspiration pneumonia [10]. Robbins et al. reported that patients with dementia or Parkinson’s disease who were instructed to drink thickened water with high viscosity for three months had a higher incidence of aspiration pneumonia during the study period compared to those instructed to drink thickened water with low viscosity for the same duration [11]. This suggests that the ingestion of thickened water with high viscosity may increase pharyngeal residue, leading to prolonged aspiration [12].

Moreover, the development of aspiration pneumonia is influenced not only by the presence or absence of aspiration but also by the ability to discharge aspirated foreign objects out of the trachea. Araie et al. reported that, in mice, the aspiration of thickened saline with high viscosity resulted in a delayed discharge of aspiration in the lungs, predisposing the subjects to lung injury, compared to the aspiration of saline without thickener [13]. From this, it is considered that in the case of aspiration of thickened water with high viscosity, stagnation of aspirated objects in the lungs may be more likely to cause lung damage and inflammation than in the case of aspiration of thickened water with low viscosity, in the background of a report by Robbins et al. that “pneumonia incidence increases with high viscosity.” Additionally, the hypothesis was proposed that water thickened to a high viscosity might hinder discharge not only from the lungs but also from the respiratory tract.

Human body discharges aspirated objects through various functions, which include the mucociliary transport function of the respiratory tract. It is an action of ciliary cells to attempt to eliminate foreign objects in the respiratory tract mucosa toward the pharynx [14, 15], and it is an important defense mechanism of the respiratory tract when the host aspirates a foreign object [16]. No studies have focused on the relationship between the mucociliary transport function and thickening agents until now. In Kartagener syndrome and primary ciliary dyskinesia, characterized by ciliary transport dysfunction, treating refractory rhinosinusitis and recurrent pneumonia is challenging. This suggests that mucociliary transport function plays a role in preventing pneumonia [17, 18].

This study, focused on clarifying the effects of the use and properties of thickening agents on mucociliary transport function, examined the relationship between two typical types of commonly used thickening agents and mucociliary transport function. This examination was conducted through the saccharine dye test, a method for evaluating mucociliary transport function [19, 20]. It is clinically essential to examine whether thickened water used for preventing aspiration can reduce airway clearance and compromise swallowing safety when aspirated.

## Methods

### Study Design and Subjects

The subjects included 23 healthy adults (7 males and 16 females with an average age of 31.3 ± 6.0 years) who consented to participate in this study. The gender difference was not taken into account because it has been reported that there is no difference in mucociliary function between men and women [21]. Those who had a cold or acute respiratory illness within 2 weeks of the study, those with a history of chronic rhinitis or chronic sinusitis, those with anatomical malformations of the nose, and those who had been diagnosed with dysphagia in the past were not included in the study. Those with a history of asthma or chronic obstructive pulmonary disease, smokers, and those taking drugs, which may be associated with an impairment of the cilia-mucus interaction, were also excluded.

The human rights of subjects were protected, and their personal information was managed in accordance with the ethical policies of the Osaka University Graduate School of Dentistry ethics committee (Approval Number: H25-E26, Date of Approval: June 2, 2017). Informed consent was obtained in writing from all participants. The protocol for this study is registered in the UMIN Clinical Trials Registry (Approval number: UMIN000046422, Date of Approval: December 21, 2021).

### Procedures

#### Preparation of Test Materials

In this study, nectar-like thickened water (with low viscosity) and honey-like thickened water (with high viscosity) were prepared using a starch-based thickening agent (Thick&Easy®, Hormel) or xanthan-gum-based thickening agent (Thicken Up Clear ®, Nestle), which are frequently used in clinical practice. This resulted in a total of four types of thickened water samples. The nectar-like and honey-like thickened water samples were prepared according to the methods specified by the manufacturer (Table 1).

**Table 1:** Thickened water preparation

Thick & Easy®, Hormel
(1) Nectar: Ion-exchange water 120 ml + 1 TBSP (15 ml) (2) Honey: Ion-exchange water 120 ml + 1.5 TBSP (22.5 ml)
NOTE: 1 TBSP = approx. 4.44 g
Thicken Up®, Nestle
(1) Nectar: Ion-exchange water 100 ml + 1 enclosed measuring spoon (2) Honey: Ion-exchange water 100 ml + 2 enclosed measuring spoons
NOTE: 1 enclosed measuring spoon = approx. 1.22 g

Next, 3 mg of saccharin and 8 mg of colorant (green food dye, Kyoritsu Foods Co., Ltd.) were dissolved in 1 ml of purified water or one of the four types of thickened water samples, resulting in five types of saccharin solutions: (1) a solution without a thickening agent, (2) a starch-based nectar-like solution, (3) a starch-based honey-like solution, (4) a xanthan-gum-based nectar-like solution, and (5) a xanthan-gum-based honey-like solution. The viscosity was 102 mPa/s for the starch-based nectar, 299 mPa/s for the starch-based honey, 213 mPa/s for the xanthan-gum-based nectar, and 435 mPa/s for the xanthan-gum-based honey. The viscosity was measured using a viscometer “VISCOMETER TVB-10 (TOKI SANGYO)ˮ. The solutions were placed in a 200 ml tall beaker and stored in an incubator at 20 °C for 1 h before measurement. The measurement conditions included a rotational speed of 12 rpm and a spindle of M3 for 30 s. However, for starch-based nectar, spindle M2 was exclusively used. In addition, the solutions were colored with the colorant to verify that the saccharin solutions were placed in the specified position.

#### Evaluation of the Mucociliary Transport Function

Referring to the established method of the saccharine dye test, mucociliary transport function was evaluated in the following manner: First, the subjects were instructed to rest in a sitting position for 30 min under a steady environment (indoors with a temperature of 22–28 °C and humidity of 40–60%). After resting, the subjects were instructed to blow their nose. Using a micropipette; the experimenter placed 1.25 μl of saccharin solution on the nasal septal mucosa near the anterior margin of the inferior turbinate of the subjects. The subjects were instructed to report when they became aware of the sweetness, which would occur when the saccharin solution reached the pharynx by ciliary transport. The experimenter measured the time from the placement of the saccharin solution to the reporting by the subjects (termed saccharin time). During measurement, in a state where the subjects were prohibited from bending their neck part backward and forward, keeping the head level, subjects were instructed to rest in a sitting position while maintaining natural breathing without eating, drinking, blowing their nose, sniffling, or coughing. The test was terminated if the subject did not become aware of the sweetness within 60 min after the start of the test, and the saccharin time of the subject was recorded to be 60 min, which was then used as the value for statistical processing.

The subjects were asked to participate in the study for any three days during the study period. The saccharin time was measured using one type of saccharin solution on the first day and two types on each of the second and third days. At the time of each measurement, one of five types of saccharin solutions was randomly assigned so that all the five types would be tested individually.

On days 2 and 3, measurements were made once in the morning and once in the afternoon. A second measurement was performed at an interval of 6 h or more to ensure that the saccharin used the first time was not tasted. Before each measurement, the subjects were instructed to blow their nose to clean the nasal cavity.

### Statistical Methods

The saccharin times were compared between thickened water samples with different viscosities and between thickened water samples with varying types of thickening agents. The Wilcoxon signed-rank test was used for statistical analysis. Bonferroni correction was additionally used for the comparison of multiple groups. The statistical significance level was set to 1%.

## Results

The saccharin times of each subject with different test materials are shown in Table 2. One subject had a saccharin time of over 60 min in the measurement using xanthan-gum-based honey. The average saccharin times were (1) 8.6 ± 4.6 min for the solution without the thickening agent (L), (2) 14.5 ± 9.0 min for the starch-based nectar (SN) solution, (3) 20.2 ± 11.3 min for the starch-based honey (SH) solution, (4) 16.8 ± 9.2 min for the xanthan-gum-based nectar (XN) solution, and (5) 23.6 ± 15.4 min for the xanthan-gum-based honey (XH) solution.

**Table 2:** Results of each saccharin time

	Saccharin time (min)		P-value					
			Between viscosities				Between thickening agents	
	Mean	SD	[in St]		[in Xa]		[in Ne]	[in Ho]
No thickening agent (L)	8.6	4.6	ref	–	ref	–	–	–
Starch-based								
Nectar (SN)	14.5	9.0	0.004	ref	–	–	ref	–
Honey (SH)	20.2	11.3	< 0.001	0.005	–	–	–	ref
Xanthan-gum-based								
Nectar (XN)	16.8	9.2	–	–	0.002	ref	0.224	–
Honey (XH)	23.6	15.4	–	–	< 0.001	0.007	–	0.615

*St* Starch-based, *Xa* Xanthan-gum-based, *Ne* Nectar, *Ho* Honey, *ref* reference, *SD* standard deviation

### Comparison Between Thickened Solutions with Different Viscosities

**Fig. 1:**
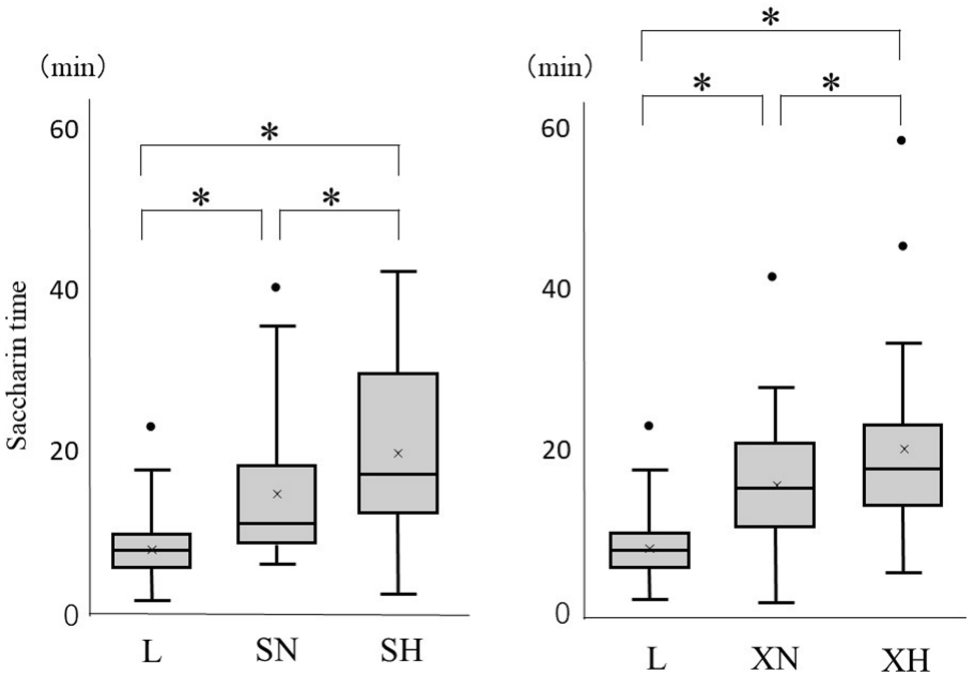
Comparison of saccharin time between thickened solutions with different viscosities. *L* no thickening agent, *SN* starch-based nectar, *SH* starch-based honey, *XN* xanthan gum-based nectar, and *XH* xanthan gum-based honey. (*:* p* < 0.01).  × represents the average value for each item. The lower whiskers represent the first quartile, and the upper whiskers represent the third quartile

The average saccharin time they followed the descending order of L < SN < XN < SH < XH. This indicates that the saccharin time tended to be longer with higher viscosity. The saccharin times of all the thickened solutions (SN, XN, SH, and XH groups) were significantly longer than that of the solution without the thickening agent (L group) (*p* < 0.01). In addition, the saccharin time in more viscous solutions was significantly longer than those in less viscous solutions, wherein the same thickening agent was used (Fig. 1).

Saccharin times were compared among L, SN, and SH groups (diagram on the left) and among L, XN, and XH groups (diagram on the right). The saccharin times of all thickened waters (SN, XN, SH, XH) were significantly longer compared to L (*p* < 0.01). In addition, saccharin times were substantially longer in SH than in SN (*p* < 0.01), as well as in XH than in XN (*p* < 0.01).

### Comparison Between Thickened Solutions with Different Types of Thickening Agents

**Fig. 2:**
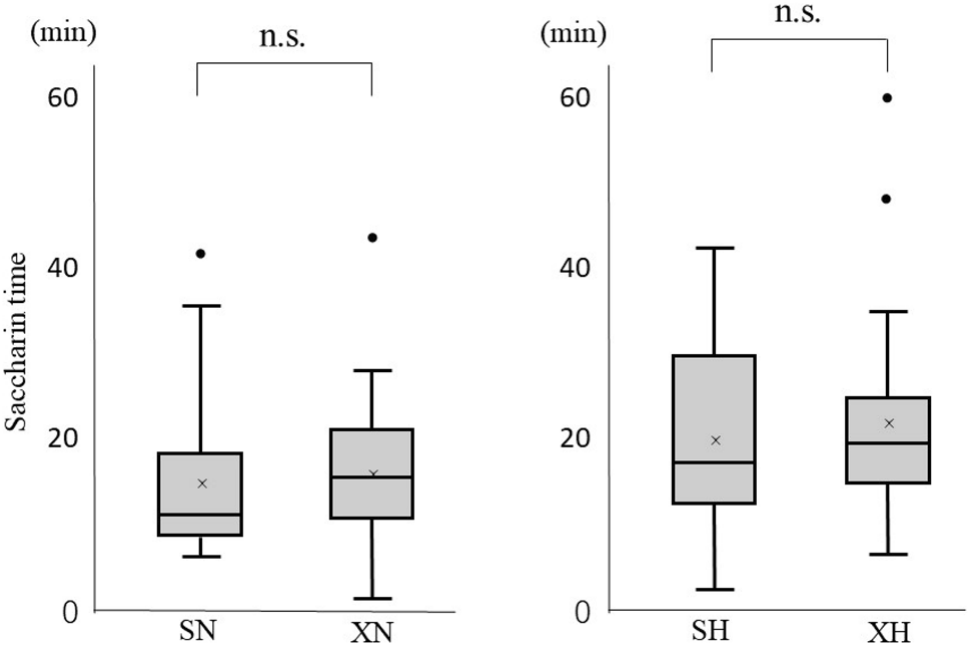
Comparison of saccharin time between thickened solutions with different types of thickening agent. *SN* starch-based nectar, *SH* starch-based honey, *XN* xanthan gum-based nectar, *XH* xanthan gum-based honey (*n.s.* not significant)

No significant difference in saccharin time was observed between the thickened solutions with the same viscosities and those with different types of thickening agents (between SN and XN groups and between SH and XH groups) **(**Fig. 2).

## Discussion

This study examined the effects of various types of thickening agents and the viscosities of thickened water samples on mucociliary transport function. The results revealed that the thickened water samples with high viscosity may take longer to be discharged via the mucociliary transport function. At the same time, there is no difference in the time of discharge between the thickened water samples with different types of thickening agents.

Aspirated objects are discharged by expectoration with coughing, which is the defensive reflex of the body, followed by ciliary transport in the trachea [14, 15, 22, 23]. However, aspirated water, which runs down the tracheal wall, is less susceptible to the wind pressure of coughing, and the mucociliary transport function is extensively involved in its discharge. It has been reported that differences in viscosity affect the ease of discharge of nasal mucus and sputum from the nasal cavity and trachea by ciliary transport [24]. Although ciliary transport may be altered by the thickening of water and the viscosity of thickened water, it has not been previously clarified. Therefore, this study examined the effects of thickening, the differences in types of thickening agents, and the viscosities of thickened water on the mucociliary transport function.

In this study, mucociliary transport function was measured using the saccharine dye test. The methods of measuring the mucociliary transport function include the observation of ciliary transport with a γ-camera using ^99m^ technetium (^99m^ Tc) as a marker [25]. Although this method is advantageous in that it can visually confirm the discharge by mucociliary transport, it exposes subjects to radioactive substances, and the facilities that have the necessary equipment for the measurement are limited. On the other hand, the saccharine dye test used in the present study is a screening test for the mucociliary transport function of the nasal cavity that has the characteristics of low harmfulness and high reproducibility [20]. Given the reported correlation between mucociliary transport functions in the nasal cavity and the trachea [26], this study presumed that a longer saccharin time in the nasal cavity indicates a prolonged discharge time for aspirated water by the mucociliary transport function in the trachea.

It has been previously reported that xanthan-gum-based thickening agents prevent aspiration more effectively than starch-based thickening agents [27, 28]. This may be because xanthan-gum-based thickening agents have less pharyngeal residue, resulting in the prevention of aspiration after swallowing. However, their effect on the ease of discharging aspirated foreign objects has not been elucidated, and no studies have discussed the selection of the types of thickening agents from this point of view. The results of the present study showed no significant difference in saccharin time between the two types of thickening agents. This indicates that the difference in raw materials of thickening agents does not affect the ease of discharging aspirated foreign objects by the mucociliary transport function, suggesting that xanthan-gum-based thickening agents are more advantageous in preventing aspiration pneumonia than starch-based ones.

The results of this study revealed that the ciliary transport of thickened water samples requires a longer time than that of water without a thickening agent and that a longer time is required for the ciliary transport of thickened water with higher viscosity. These results indicate that the ease of discharging aspirated foreign objects involves two factors: objects or fluids being transported and ciliary movement.

First, the shear stress of thickened water increases with increasing viscosity. Due to the increased shear stress, it requires a longer time for the thickened water itself to flow [29]. This study also suggested that the flow of thickened water is altered because of the thickening agent and the increased viscosity, requiring a longer time for ciliary transport.

Next, the ciliated epithelium of the trachea and nasal cavity has a periciliary layer between the cilia, as well as a mucous layer above it, and the two layers maintain proper hydration of the surface by supplying and accepting water [16, 30]. An increase in the concentration of the sol layer leads to the generation of partial osmotic pressure and changes the osmotic pressure of the periciliary layer. This is known to disrupt proper hydration, reducing the mucociliary transport function and prolonging the time required for removing mucus [31]. The thickened water used in the present study has a higher osmotic pressure than fresh water because of its macromolecular solvent. Therefore, an increase in the concentration of the sol layer due to the thickened water might have altered the osmotic pressure and lowered the mucociliary transport function, affecting the time required for ciliary transport.

Robbins et al. reported a higher incidence of pneumonia in patients using thickened water with high viscosity. This study contributes to clarifying the cause behind their observation. Moreover, this study found that there is a paradox in which while the use of thickened water aims to prevent aspiration [7], its discharge may be difficult when it is aspirated.

It has been reported in recent years that taking drugs with thickened water may adversely affect their absorption [32, 33]. Based on this and the results of the present study, it may be necessary to review the advantages and disadvantages of the use of thickening agents.

The saccharine dye test used in this study is a screening test for the mucociliary transport function of the nasal cavity, and it does not directly observe the ciliary transport in the trachea. Therefore, the lack of direct observation of the trachea is considered a significant limitation in the examination of the discharge from the trachea. However, a correlation between mucociliary transport functions in the nasal cavity and trachea has been established [26, 34]. In conditions like Kartagener syndrome and primary ciliary dyskinesia, characterized by ciliary transport dysfunction, clinical evidence shows simultaneous reduction or promotion of mucociliary transport function in both the nasal cavity and the trachea [17, 18]. Therefore, measuring saccharin time in the nasal cavity seems to be able to reflect the effect of thickening agents on the mucociliary transport function in the trachea.

### Clinical Implications

- Thickened water with higher viscosity may be more challenging to be discharged by the mucociliary transport function.
- The use of thickening agents to prevent aspiration and the difficulty of discharging aspirated thickened water must be weighed against one another in patients with a high risk of aspiration and weak coughing reflex or impaired airway clearance.
- While the use of thickened water aims to prevent aspiration, its discharge may be difficult when it is aspirated.

## Conclusion

Thickening agents have often been used as a “means of preventing aspiration.” However, it had been unclear how different thickening agents would affect the discharge of aspirated, thickened water from the trachea. This study, focusing on aspirated thickened water samples, examined the effects of different types of thickening agents on mucociliary transport function. The results of this study indicated that thickened water with high viscosity may be more challenging to discharge by the mucociliary transport function. Therefore, the use of thickening agents to prevent aspiration and the difficulty of discharging aspirated thickened water must be weighed against one another in patients with a high risk of aspiration, weak coughing reflex, or impaired airway clearance.

## Data Availability

The data that support the findings of this study are available from the corresponding author, K. N, upon reasonable request.

## Declarations

### Clinical Research Registry

The protocol for this study is registered in the UMIN Clinical Trials Registry (Approval number: UMIN000046422, Date of Approval: December 21, 2021).
